# Research Progress on Skid Resistance of Basic Oxygen Furnace (BOF) Slag Asphalt Mixtures

**DOI:** 10.3390/ma13092169

**Published:** 2020-05-08

**Authors:** Song Li, Rui Xiong, Jiahui Zhai, Kaiyin Zhang, Wenyu Jiang, Fa Yang, Xiaoquan Yang, Hua Zhao

**Affiliations:** 1 School of Transportation, Wuhan University of Technology, Wuhan 430063, China; ls18229013710@163.com (S.L.); zhangky1960@126.com (K.Z.); 2 School of Materials Science and Engineering, Chang’an University, Xi’an 710061, China; 2017131023@chd.edu.cn; 3 Yunnan Communications Investment & Construction Group Co., Ltd., Kunming 650228, China; 2019231019@chd.edu.cn; 4 Road Network Monitoring and Emergency Command Center of Yunnan Provincial Department of Transportation, Kunming 650200, China; xqyang456@163.com; 5 School of Civil Engineering and Architecture, Nanchang University, Nanchang, 330031, China; zhaohua@ncu.edu.cn

**Keywords:** road engineering, asphalt mixtures, BOF slag, skid resistance, differential polishing principle

## Abstract

In order to ensure the safety of traffic, asphalt pavement is commonly required to utilize aggregates with excellent anti-abrasion property. This results in the lack of high-quality aggregates. The incorporation of solid waste in the aggregates is regarded as a high potential alternative for solving this problem. Since its material properties, such as rough surface, high Polished Stone Value (PSV) and the excellent adhesion property of asphalt, Basic Oxygen Furnace (BOF) slag can effectively improve the skid resistance of asphalt mixtures. First, the material properties of BOF slag are reviewed in this study. Then, the skid resistance of asphalt mixtures and aggregates are commendably evaluated by the Polished Stone Value test, Wehner/Schulze Tester, Aachen Polishing Machine, British Pendulum Test and Sand Patch test. The physical and mechanical properties of BOF slag play a key role in asphalt mixtures. This review found that the skid resistance mechanism of the BOF slag asphalt mixture is governed by factors such as BOF slag properties, incorporation methods and gradation types. Finally, the economic and environmental benefits of BOF slag asphalt mixtures were discussed. In addition, the function of gas catalysis and the melting of ice and snow can be added to the BOF slag asphalt mixture for a cleaner development in engineering. Furthermore, the existing problems, research directions and corresponding measures in this field are directed towards more durable and functional asphalt pavement construction.

## 1. Introduction

The skid resistance of asphalt pavement is not only crucial for traffic safety, but also an important aspect of asphalt pavement durability [1,2,3]. Asphalt pavement is composed of asphalt, mineral aggregates and filler. Mineral aggregates, which have a great impact on the anti-skid of asphalt pavement, consist of more than 70% by weight in asphalt mixtures [4,5]. In order to ensure the safety of traffic, the pavement surface is generally required to utilize aggregates with excellent anti-wear and anti-abrasion properties. This requirement imposes huge pressure on the store of high-quality natural aggregates [6,7,8,9,10]. To preserve natural resources, researchers began to look for materials that can replace natural aggregates.

The Basic Oxygen Furnace (BOF) slag, main solid waste of steel industry, has high Polished Stone Value (PSV) and wear resistance due to containing significant amounts of iron. These make it possible to use the BOF slag as aggregates in surface layers to elevate the skid resistance [11]. Two thousand years ago, the Roman Empire used slags in road-based construction; in the nineteenth century, America and England also applied slag to road-based material and rail ballast [12,13]. Recently, Bessa et al. found that the main anti-slip parameters of BOF slag, such as polished value and abrasion value, are superior to natural aggregates [14]. This is related to iron and manganese ions in the steel slag. These ions have a strong polarization ability, which helps to break the original orthosilicate structure and form a new large and complex silico-oxygen group. Hence, BOF slag has excellent abrasion resistance, compression resistance, anti-skid and other properties [15]. The Transport Research Laboratory (TRL) points out that when the PSV value of steel slag is higher than 60, it can be used in the wearing courses [16]. Emery applied BOF slag in dense gradation asphalt mixtures and open gradation asphalt mixtures to ensure the safety of the asphalt pavement [17,18,19]. Asi attained that asphalt mixture containing 30% slag has a superior skid resistance performance than traditional mixture [20]. In addition, the steel slag asphalt mixtures have superior skid resistance performance compared to natural aggregates used in the surface layers [21]. On the whole, the incorporation of BOF slag in asphalt mixtures is a promising way to settle aggregate insufficiency and diminish environmental depredation.

This review documented the mechanism of skid resistance of BOF slag asphalt mixtures, summarized the effects of BOF properties, incorporation methods and grading types on skid resistance, and discussed the skid resistance evaluation methods of asphalt mixtures and aggregates. Additionally, it further highlighted some main challenges and considerations in order to provide guidelines for selecting the appropriate BOF slag to enhance the anti-skid of asphalt mixtures and the utilization of BOF slag.

## 2. Materials

### 2.1. Chemical and Mineral Composition of BOF Slag

Influenced by different steelmaking processes, the chemical composition of steel slags mainly consists of elements such as calcium, iron, silicon, magnesium, aluminum, manganese, and phosphorus. The chemical composition of BOF Steel from different areas varies, which is presented in Table 1. Generally speaking, the chemical composition of materials can be gained by X-ray Fluorescence (XRF). Table 2 shows XRF test results of different aggregates from China. The following is a brief introduction of China, as an example.

According to Table 2, iron oxides (FeO, Fe_2_O_3_), lime (CaO), silica (SiO_2_), and alumina (Al_2_O_3_) are the main chemical constituents of steel slags, which are quite different from natural aggregates such as limestone and basalt. Generally speaking, high SiO_2_ content means high hardness, and benefits to the mineral skeleton; as for Al_2_O_3_, it is good for adhesion between aggregate and asphalt. However, influenced by steelmaking process, the f-CaO is inevitably produced, and this will bring some problems. For example, free calcium oxide reacts with water, turns into calcium hydroxide Ca(OH)_2_, and damages the pavement structure.

Mineral composition refers mainly to calcium silicate phases (C_3_S, C_2_S), and RO phases, as shown in Table 3. These mineral phases relate to the properties of BOF slag as coarse aggregate in asphalt mixtures, especially as polishing properties and volume instability behavior.

### 2.2. Physical and Mechanical Properties of BOF Slag

**Figure 1:**
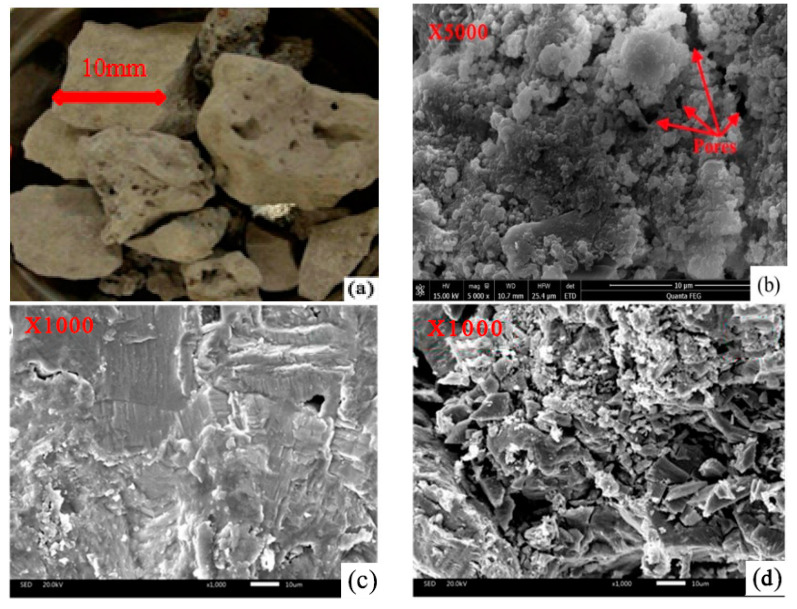
Macro and micro morphology of BOF slag. (**a**) is macro morphology; (**b**–**d**) are micro morphology.

**Figure 2:**
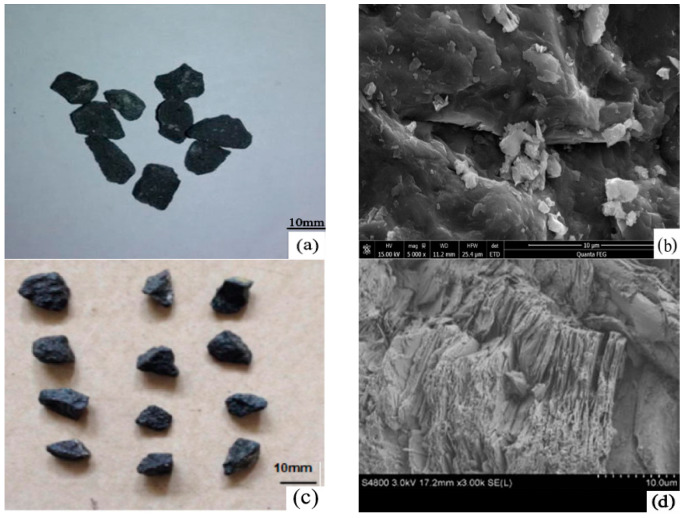
Macro and micro morphology of basalt and limestone: (**a**,**b**) basalt; (**c**,**d**) limestone.

BOF Slag is an aggregate that is rough, porous, and rounded, as shown in Figure 1. Figure 2 indicates the macro and micro morphology of basalt and steel slag; and Figure 2a,b is basalt; Figure 2c,d is limestone. There is evidence of differences of surface textures between basalt and steel slags. Compared with basalt, the rougher and more porous surface of steel slag increases its adhesion with asphalt [26,27,28,29].

Table 4 shows a difference between the properties of BOF slag and traditional aggregates. It is concluded that the steel slag has higher density. This helps to elevate the abrasion but also causes the segregation between BOF slags and natural aggregates in the transportation. The segregation includes material segregation and temperature segregation. Controlling stack height in the truck can avoid material segregation to some extent. Temperature segregation is related to the cover of the truck.

As it is known, these indicators reflect the anti-skid of asphalt mixtures, the LA and PSV values are applied to estimate the properties of aggregates. The abrasion value of the aggregate that is estimated by the abrasion test represents the mass loss of the aggregate in the abrasion process, and can show the durability of asphalt pavements. The polishing property of aggregates describes the process of aggregate microtextures being gradually “cut”. Generally speaking, only when the PSV value is more than 50 and the LA value is less than 20, can the aggregates be used in anti-slip layers. These requirements ensure enough microtexture of aggregates after suffering from repeated traffic loadings. BOF slag contained significant amounts of iron, which helps to break the original orthosilicate structure and form a new large and complex silico-oxygen group. Hence, the PSV of BOF Steel is higher. As for the Crush value, the BOF slag is the smallest in these aggregates. This indicated that BOF slag aggregate has superior resistance to crushing, and an internal friction angle, as well as better performance in road pavements than natural stone aggregates.

**Figure 3:**
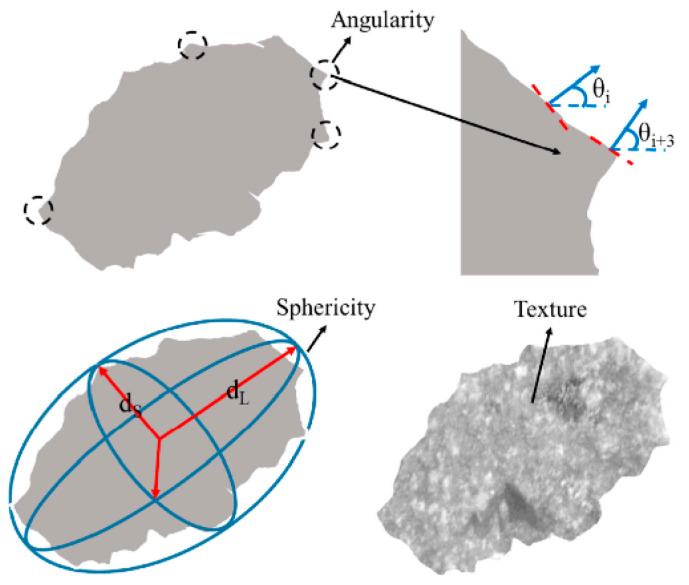
The morphological characteristics of aggregates.

The texture of asphalt pavement consists of microtextures and macrotextures. Macrotexture is closely associated with gradation of aggregates, while microtexture is affected by morphological properties of aggregate. The morphological characteristics of aggregates can be divided into three dimensions, namely surface texture, shape and angular, such as Figure 3, which has a great influence on the skid resistance of asphalt pavements [31,32,33]. Recently, the Aggregate Imaging System (AIMS) was applied to evaluate the coarse aggregate’s morphological characteristics in order to improve the accuracy.

When the texture of the aggregate surface is rough, it is beneficial to improve the contact area between the asphalt and aggregate and enhance the adhesion. The shape and angle attach the importance to the formation of the structure of interlock. Due to a lower length-to-particle ratio and a smaller number of needle-like particles, the steel slags easily form stable skeleton structures. However, a higher angle of BOF slag does harm to the asphalt mixture’s anti-rutting performance, and should be considered. In conclusion, due to the high contents of SiO_2_ and Al_2_O_3_, the BOF slag has excellent PSV, resistance to wear and polish. This makes it appropriate for incorporating in asphalt mixtures as coarse aggregate to improve the performance of skid resistance.

### 2.3. The Volumetric Expansion of BOF Slag

As showed in Section 2.1, the steel has the potential possibility of swelling, due to it containing f-CaO. This is harmful to the durability of asphalt mixtures. The durability of asphalt mixtures, referring to the ability to withstand various loads, including vehicle and environment loads, is important. To ensure the durability of asphalt mixtures, the expansion of the steel slag must be taken into consideration [34]. There are three methods to improve the stability of steel slags, as shown in Table 5. According to the process principle, the three ways can be divided into two types—active and passive. Evidently, aging is a passive method. Chen et al. [35] used this method to enhance the stability of steel slags, and the results showed that the stability of steel slags aged to meet specification requirements. In this way, the steel slag was moved to a vibrating–dewatering screen and washed by a vertical high-pressure water jet. However, this traditional testing method is not only time-consuming, but also damaging to the environment. Consequently, Chen et al. applied a hydration and silicone resin to cover the steel [36]. This worked as follows: first, the fully hydrated BOF slag (FHS) was immersed in silicone resin for 1 h; then, FHS covered with silicone resin was cured in the oven at 60 °C for 24 h. The process needed to be repeated for three cycles, and BOF slag modification of hydration and silicone resin (HSS) was then obtained. The results demonstrated that the volume expansion ratio of Newly Crushed BOF Slag (NCS) was 5.1 and 7.7 times the size that of FHS and HSS.

Ma et al. [44] applied this way of covering a steel slag surface to elevate the performance of steel slag asphalt mixtures. The results indicated that this way of using a silicone waterproof agent proved to be the best treatment effect. The expansion ratio reduced by 34% and the dynamic stability increased by 20%. This indicated that covering steel slag surfaces can elevate the performances of steel slag asphalt mixtures.

Crisman et al. [45] established a model to predict the long-term skid resistance of pavements containing natural aggregates and steel slags. The skid resistance of the pavement was measured by British Pendulum Number (BPN), and the Polished Stone Value (PSV) was applied to estimate the aggregate’s anti-polishing performance. The aggregates of wearing course was blended by slag (60%) and limestone (40%). During an eight-year period, the British Pendulum tests were used in the different areas. The PSV was determined by the standard UNI EN 1097-8. An analysis between the anti-skid performance of the in-service pavement and PSV test of the aggregate can be expressed by Equations (1) and (2).

(1) $$t\;≌\;(\frac{T}{2520})$$

(2) $$\mathrm{BPN}(T)=(\frac{A\cdot 7\cdot v_{\mathrm{CLA}}}{T}){}^{4}+\frac{B\cdot \mathrm{PSV}}{k}$$

where A and B are two regression parameters; v_CLA_ is the speed in rounds per minute (360 RPM); T is the number of car; PSV is the value that comply with EN 1097-8; t is the polishing time (s); k is a coefficient that considering of difference between the pavement and laboratory test.

The results showed that the slags had a superior long-term skid resistance—namely more durability—than basalt.

## 3. Evaluation Methods of Anti-Skid Performance

Asphalt mixture is composed of aggregate and asphalt, and aggregate is used as the direct contact medium with tires. Therefore, for sufficient skid resistance of the road surface, coarse aggregates of asphalt mixtures should be correctly selected. The British Pendulum Tester, Polished Stone Value test, Wehner/Schulze test and other tests are currently in use for selecting aggregates and evaluating asphalt mixtures. Water, the polishing agent, and affinity of the aggregate have vital impact on the skid resistance development of the specimen, namely the removal speed of the bitumen film. The following Table 6 summarizes the characteristics of them.

### 3.1. Polished Stone Value Test (PSV)

The Polished Stone Value test (PSV) is widely used to evaluate the polishing property of aggregates. The PSV test is only applied to estimate the skid resistance of surface layer aggregates. The specimen can be polished by two stages. In the first 3 h, the specimen is subjected to cleaning on the surface. The second 3 h is related to the polishing action. One used coarse emery and the other used fine emery. The degree of polish of the specimens was then obtained by the British Pendulum device of the F-scale, and the method was recorded in BS EN 13036. The PSV coefficient is calculated by Equation (3) [46].

However, PSV in correspondence with the BS EN 1097 test method does not correspond with the PV-10 measured correspondence with AASHTO T279 method on occasion [47]. Moreover, it is difficult to effectively simulate the influence of the roll pneumatic rubber tires on the pavement.

PSV = S + (52.5 − C)(3)

where PSV is the polished stone value of aggregate British Pendulum Number (BPN), S is the average value of four test specimens, BPN; C is the average value of four standard specimens, BPN.

### 3.2. The British Pendulum Tester (BPT)

The British Pendulum Tester has been universally applied to measure the skid resistance of aggregates and asphalt mixtures respectively, according to EN1097-8 and NLT-175/98. The Pendulum Test evaluates low-speed friction (about 10 km/h). In the experiment, the pendulum with a rubber slide is released at a certain position. By recording the position where the pendulum rises after rubbing against sample surface, the British pendulum number (BPN) can be obtained. The friction is related to a reduction in the length of the oscillation. It is acknowledged that the British Pendulum Tester is an empirical test. Fwa et al. [48] developed a finite-element simulation model to calculate the corresponding of BPN value with the coefficient of friction, and the results indicated that the measured BPN and the friction coefficient have a unique one-to-one mechanistic relationship.

In addition, The BPT can effectively identify the direction of skid resistance in a polished pavement [49].

### 3.3. The Sand Patch Test

The Sand Patch Test can be applied to determine the text depth of asphalt mixtures. In the program, a graduate cylinder filling with 25 mL standard sand was needed. The size of standard sand particle ranged from 0.15 mm to 0.30 mm. The sand was then spread on the central area of the samples as a circle. The diameter of the sand patch needed to be measured [50], and the mean texture depth (MTD) can be calculated by Equation (4) [51].

It is worth noting that the Sand Patch result is affected by the gradation of asphalt mixtures. Generally speaking, open gradation provides a superior result.

MTD = 4V/πD^2^ (4)

where the MTD is mean texture depth, mm; V represents the volume of sand introduced, mm^3^; and the D is average diameter of the circle, mm.

### 3.4. W/S Tester

To simulate a real situation of asphalt pavements, the rubber tires were used to polish the samples in the Wehner/Schulze (W/S) tester. The W/S device has two positions, one is used to polish, the other for measuring friction. Aggregates or asphalt mixture samples can be evaluated [52]. The polishing station with three rubber cones can be lowered and move across the specimen surface at a predefined loading. The rotation speed of the ring is 500 r/min, which is equal to the linear velocity of 17 km/h. The contact load between the rubber cones and specimen is 0.4 MPa. The slip ratio between the rubber block and specimen is about 0.5% to 1%, which is close to the slip ratio of the rolling rubber tire to road surface. However, it cannot change polishing agent to adapt different situations [53].

### 3.5. Aachen Polishing Machine Polishing Machine (APM)

The Aachen Polishing Machine (APM), designed by RWTH-Aachen University, used genuine car tires (Type: Vanco-8,165/75 R 14 C 8PR 97/95 R TL from Continental) to polish the specimen. The APM tester can be used to measure not only the dynamic friction of aggregate but also the asphalt mixture specimen. The inner pressure and loading are 0.2 MPa and 200 kg, respectively. Generally speaking, polishing agent and water are spread on the surface at a rate about 27 ± 7 g/min, and the polishing time was controlled to 300 min. Wang et al. [45,54,55,56] applied the APM tester and W/S Tester to estimate the effect of the polishing conditions on the anti-skid. The results demonstrated that the difference of the sand in the summer and winter could lead to different results.

**Table 6:** Polishing property test method of aggregates and asphalt mixtures [57,58,59,60,61].

Methods	Materials	Standards	Indicators	Advantages	Disadvantages
Polished Stone Value test (PSV)	Aggregates	EN 1097-8	PSV	Simple	Lower accuracy
Wehner/Schulze Tester	Aggregates/mixtures	EN 12697-49	FAP *	Ability to simulated the interaction between tire and road surface	Certain conditions
Aachen Polishing Machine	Aggregates/mixtures	/	PSV	Good correlation with the reality of the road	Certain temperature
The British Pendulum Tester	Aggregates/mixtures	EN 1097-8	PTV	High efficiency	Lower accuracy
Sand Patch test	Mixtures	EN 13036-1	MTD	Simple	Lower accuracy

* FAP is Friction after Polishing; PTV is Pendulum Test; MTD is Mean Texture Depth.

## 4. Key Factors Influenced Performance of BOF Slag Asphalt Mixtures

### 4.1. BOF Slag Properties

Skid resistance is one of main functions of asphalt pavements. When the pavement was exposed to the wheel load, the surface texture could not maintain its original shape and the skid resistance of asphalt pavement decreased. It is usually considered that this process is associated with coarse aggregates, namely, mechanical properties, and adhesion properties. The BOF Slag had relatively excellent adhesion and polishing properties, and improved the anti-resistance property of the asphalt mixtures when it was added into the asphalt mixtures.

#### 4.1.1. Physical and Mechanical Properties of BOF Slag

Bessa et al. [14] used the Los Angeles abrasion device and the AIMS to evaluate angularity and the surface texture’s change of steel slag and natural aggregates. Compared with the natural aggregates, the steel slag showed a lower loss of surface texture and low roughness. As for angularity, the steel slag had the lowest increase on sphericity, but still kept the highest average value after the degradation when compared with the other aggregates. The results indicate that BOF slag is the most resistant to polish. Vaiana et al. assessed the anti-skid of the steel slag with the British pendulum test. The result of the BPN changed before and after polishing, with a mean value of about −7%, 1/3–1/2 of kinzigite and limestone. It also showed a superior resistance to polish [62]. In addition, the high contents of SiO_2_ and Al_2_O_3_ have positive influences on hardness [8]. Additionally, the BOF slag can provide a high PSV value though the different polishing effect between the different mineralogies.

#### 4.1.2. The Adhesion Properties of Steel Slag with Asphalt

The adhesion of the steel slag with asphalt is crucial for the durability of skid resistance. The adhesion ability between asphalt and aggregate is composed of physical and chemical adhesion. The physical adhesion refers to the surface adsorption, tension, energy, and so on, which are related to the morphology of surface. The chemical adhesion mostly depends on the chemical reaction between asphalt and aggregate. It is obvious that rough surface texture, porosity and high alkalinity strengthen the adhesion ability.

Xiao et al. [63] designed an Active Adhesion Evaluation Method (AAEM) that can reduce the operation error in the boiling process, and transform it into a dependable operation. Subsequently, the AAEM and the digital image processing system were used to study the adhesive properties between basalt and steel slags. In both the AAEM and boiling water methods, the adhesion performance of steel slags is better than basalt. This owes to the content of SiO_2_, which increases the alkalinity of steel slags.

Xue et al. [30] investigated the possibility of BOF slags incorporated as aggregates in asphalt mixtures. The SEM test indicated that the steel slag has rougher surface texture than natural aggregates, which strengthens their adhesion ability with asphalt. Moreover, the BPN value and Texture Depth revealed that the skid resistance of the asphalt mixture with steel slags is better.

Gao et al. [64] introduced the fractal dimension to quantify the structure of the aggregate surface, and found that the fractal dimension of the aggregate had a good positive correlation with tortuosity. This indicates that the shape in the pores of the steel slag is more complex than the diabase, and the bitumen can form a better bite force with the complex texture of the steel slag.

Shen et al. [65] studied the adhesion ability between the steel slag aggregate and rubber asphalt. A pull-out test indicated that the interfacial adhesion strength between the steel slag aggregate and rubber asphalt is larger than the diabase. Moreover, the SEM test revealed that the amount of pores on the surface of steel slags enhance embedding depth.

Liu et al. studied the reaction of the steel slag with the asphalt interface. The mineral composition and structural composition of the steel slag were tested by X-ray diffraction (XRD) and Fourier transform infrared spectroscopy (FTIR), respectively [66]. The results indicate that the steel slag contained some calcium hydroxide, and can react with asphalt to improve the adhesion between the steel slag and asphalt.

### 4.2. Incorporation Method

**Figure 4:**
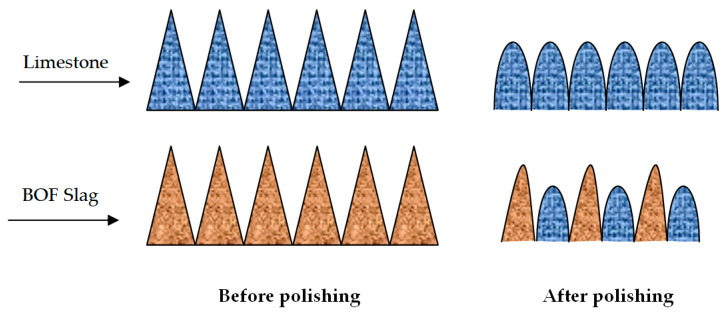
The contrast between general polishing and differential polishing.

Asphalt mixtures generally consist of asphalt, coarse aggregate, fine aggregate and mineral filler, and the performance of asphalt mixture is based on the coarse aggregates volumetric parameters. Differential polishing, namely blending aggregates which have very different intrinsic properties is beneficial to both pavement and environment. Compared with common polishing, differential polishing can utilize difference between discrepant aggregate in order to enough macrotexture, as shown in Figure 4. Generally speaking, the greater gap in mineral hardness of the different aggregate is, the better skid resistance of asphalt mixtures is.

Replacing in mass, volume and volume mass conversion method are three methods often adopted for asphalt mixtures design, and information of these are shown in Table 7. Mass substitution method refers to that the replacing aggregate is equal with the original aggregate in quality. Volume substitution method ensures the volume parameters of asphalt mixture are basically unchanged after the blending. Volumetric mass conversion method takes the aggregate mixed in proportion as a whole and carries out the mixing ratio design according to the original Marshall design process. The above methods have their own characteristics and are suitable for different situations. Due to the volume parameters of BOF slag and natural aggregates are quite different, volume parameters of asphalt mixtures must be taken into account.

Rondon-Quintana et al. [72] designed an experiment to evaluate the steel slag asphalt mixture by different replacement (in mass and volume). The results showed positive effect in the performance of the mixtures when replaced in volume. When it comes to the replacement in mass, the adhesive ability of the asphalt-aggregate was decreased.

Niu [69] applied volumetric mass conversion method to discuss the anti-skid of Stone Mastic Asphalt (SMA) steel asphalt mixtures. Results showed that the Texture Depth is 1.67 mm, the outcome of volume substitution is 1.2 mm. Both are higher than basalt asphalt mixtures. Therefore, it is more suitable to adopt the volume substitution method or volume mass conversion method. However, the porous property of BOF slag causes higher binder content, which should be considered.

However, studies on differential polishing are few, and the composition design method of incorporating BOF steel in asphalt mixture with natural aggregate is worth further exploration. In addition, BOF steel asphalt mixture’s anti-skid durability is also very important under the action of environmental factors and driving load. Incorporating BOF steel into an asphalt mixture can not only effectively solve the problem of the gradual shortage of high-quality anti-skid aggregate for roads, but also add to the economic and environmental benefits.

### 4.3. Gradation

Theoretically, BOF slag can be incorporated as an aggregate into the mixtures whether coarse or fine. Cao et al. [73] investigated the two kinds of SMA-13 asphalt concrete with the steel slag as incorporated coarse aggregate and fine aggregate respectively, and found that the oil–stone ratio of the former is 0.5% lower than the latter. Chen et al. [74] believed that, when the diameter of steel slag is less than 4.75 mm, it is easier to carry dust and increase the construction cost. Wu et al. [75] stated that the scheme of choosing the steel slag as coarse aggregate (>4.75 mm or 2.36 mm) was more economic. In addition, the steel slag asphalt mixtures have lower asphalt–aggregate ratios. Therefore, it is suitable for use as coarse aggregate in asphalt mixtures.

Wang [76] used various gradations of steel asphalt mixtures in a study on skid resistance. The optimal dosage of SMA-10 and SAC-10 are 40%, 60% respectively, where the Texture Depth value is 0.84 mm, 0.98 mm, and this value is higher than 0.55 mm (the minimum requirement of Texture Depth value).

Niu [69] studied the impact of gradations on the performance of asphalt mixtures. He compared two kinds of gradations of AC-13 and SMA-13. The results indicated that of the two properties, the gradations are different based on the BPN and Texture Depth test. The Texture Depth of SMA-13 is twice as much as AC-13, but the BPN value is close.

Chen et al. [77] used Analysis of Variance (ANOVA) to study the influence of gradation on anti-slip performance. The results showed that the grading type was thought to be not statistically significant at the 95% confidence level. It showed that the aggregate and grading types have no significant influence on BPN value. However, if Texture Depth is taken as the evaluation index, there may be different results.

Texture Depth, BPN_20_, and DF_60_ are commonly used to evaluate the anti-slip performance of asphalt mixtures. In different gradations, only Texture Depth changed clearly. Because texture depth refers to the macroscopic structure of the mixture, which is affected by gradation, gradation has no significant influence on the skid resistance of the steel slag asphalt mixture.

## 5. The Skid Resistance of BOF Slag Asphalt Mixtures

Generally speaking, resistance to skid, in terms of the British pendulum number or texture depth, is higher in the steel slag asphalt mixes. This is related to its properties as a coarse aggregate. On one hand, the coarse aggregate’s surface texture, hardness, porosity, wear resistance and polishing are crucial for the anti-slip performance. On the other hand, the steel slag is an alkaline aggregate and has a porous surface, so it has good adhesion to asphalt.

### 5.1. Macro Performance Anti-Sliding

Some studies showed that skid resistance was better in mixes that only used BOF slags as coarse aggregates. Most of the studies about these are summarized in Table 8.

Xu et al. [78] conducted research on improving skid resistance of asphalt mixtures by adding BOF slag. According to the results of the BPN and Texture Depth test, the anti-skid of asphalt mixtures could be enhanced by the addition of the steel slag. The slip resistance and structural depth of the steel slag asphalt mixtures were comparable to basalt asphalt mixtures.

Shen et al. [79] presented a survey on the skid resistance of steel slag asphalt by changing the content of the steel slag. The results of the structure depth test showed the skid resistance of asphalt mixes could be enhanced by the addition of steel asphalt. When the steel slag weighs 50% of the aggregate, the structure depth was increased by 0.05 mm compared to the net asphalt mix. However, with the steel slag content higher than 50%, the structure depth decreases, but remains better than net asphalt mixtures.

Nguyen et al. [80] measured the surface roughness and slip resistance of steel slag asphalt test section. The texture depth values demonstrated that steel asphalt mixture is higher than a common asphalt mixture.

Motz et al. [11] evaluated the use of more than 25 steel slag roads in Germany. After a long period of using, the steel slag asphalt pavement still maintained a high polish value and these roads had no harmful effect on the environment.

Chen et al. [81] estimated the skid resistance of SMA mixtures with different BOF steel size by the BPN test. In the BOF slag coarse aggregates (BSCA), content with the size of 9.5–16 mm, 4.75–9.5 mm, 4.75–16 mm accounted for 45%, 29% and 16% of the total volume of the mineral mixture, respectively. The results indicated that the BPN value of SMA mixture increased by 3.4%, 6.3% and 11.4%, respectively. It was indicated that coarse particles of BSCA played an effective role in improving the antiskid of SMA mixtures.

Asphalt mixtures containing copper tailings and EAF steel slag were studied [82]. Skid number and mean texture depth were improved by 29.3% and 80.5% respectively. It is the angular shape, the high porosity and the surface texture of copper tailings and EAF steel slag that enhance the interlocking structures and adherent bond formed in asphalt mixtures.

### 5.2. Micro Performance Anti-Sliding

**Figure 5:**
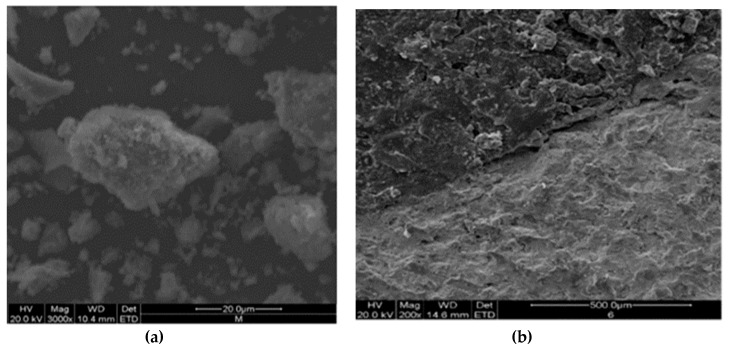
Scanning electron microscope (SEM) image of the steel slag and steel slag with asphalt interface. (**a**) The steel slag; (**b**) Steel slag–asphalt interface.

Liu et al. [66] investigated the adhesion mechanism of the steel slag with asphalt, as shown in Figure 5. The results showed that the pore structure of the steel slag is beneficial to form a certain embedding depth between the asphalt and the steel slag. This can improve the interface adhesion between asphalt and the steel slag, as well as the durability of skid resistance. Shen et al. [65] studied the adhesion property between steel slag aggregate and rubber asphalt with the pull-out test, and gained a similar conclusion.

Komg et al. [27] analyzed the effect of sphericity, gradient angularity and micro texture on BOF slag asphalt mixtures with the image measurement system (AIMS). The result showed that the extremely high angularity of BOF slag will weaken the VV of AC 13.

**Figure 6:**
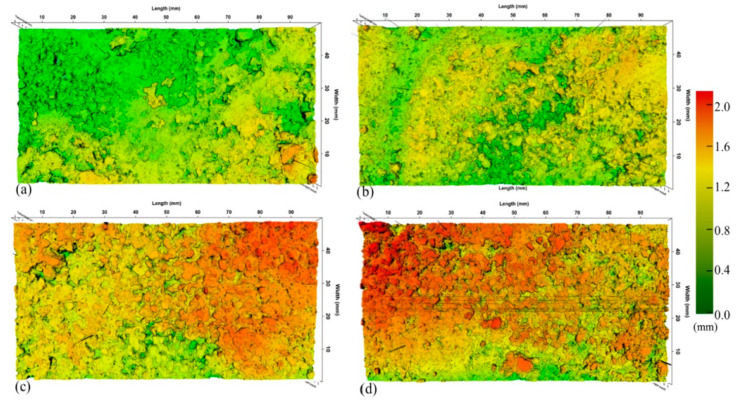
Three-dimensional surface models of different steel slag micro-surfacing, (**a**) basalt used as coarse and fine aggregates; (**b**) coarse basalt aggregates and fine steel slag; (**c**) coarse steel slag aggregates and fine basalt aggregates are; (**d**) steel slag used as coarse and fine aggregates.

Xiao et al. [22] compared the texture of different steel slag micro-surfacing by laser scanning, as shown in Figure 6. It can be found that steel slag replacing basalt used as coarse basalt has a significant positive effect on surface texture.

From the above-mentioned studies, it can be concluded that adding BOF steel significantly improves the structure of interlocks and increases the texture of asphalt mixtures, which is beneficial to the skid resistance of asphalt mixtures.

## 6. Economic and Environmental Benefits of BOF Slag Asphalt Mixtures

In 2014, the production of steel slags was estimated to be 200–223 million tons across the world. If the steel slag was not incorporated in asphalt mixtures or other fields, the general way of disposal is storage or burying. This has the potential to lead to environmental and health damage, such as water and soil pollution due to the leaching characteristics of BOF slag [83]. In particular, pH, chemical oxygen demand (COD), oxygen depletion, salinity, and metal concentrations of water were increased [84,85]. Given the high costs and constraints of disposal, the recycling and re-utilizing of BOF Steel has become significant [86,87]. In addition, the recycling of BOF Steel can produce a huge economic effect. In the United States, the steel slag used as an aggregate for pavement construction and other miscellaneous applications accounts for 50–70%, 10–15%, respectively [88]. In Europe, the consumption of BOF and other steel slags in pavement has gone from 6.6 million tons in 2000 to 65.3 million tons in 2016 [89]. In China, based on the annual output of 2.16 million tons of steel slag of the Wuhan Iron and Steel Company, researchers calculated that an economic benefit of 108 million yuan will be generated in one year [90]. In addition, the re-utilization of BOF Steel in asphalt mixtures has a potential impact on the environment due to the hazardous elements leached from BOF Steel. The heating and compacting of BOF Steel are more costly. Despite some performance challenges, the incorporation of BOF Steel into asphalt mixtures indicates potential benefits to not only the environment, but also the economy.

## 7. Conclusions and Future Developments

BOF slag is widely applied in asphalt pavement engineering to improve the skid resistance due to its excellent mechanical strength and adhesion ability. This review first introduces material characteristics of BOF slag asphalt mixtures and the polishing property testing methods of aggregates and mixtures. Meanwhile, the composition design of BOF slag asphalt mixtures is summarized, including BOF properties, incorporation methods and gradation. Finally, the economic and environmental benefits of BOF Slag asphalt mixtures were discussed. Based on these studies, the following conclusions can be obtained:

1. The skid resistance of BOF slag asphalt mixtures seems to be dependent upon physical and mechanical properties of the BOF slag. The high density, angular shape and the irregularities on the BOF slag aggregate’s surfaces ensure substantial resistance to deterioration under construction and continuous traffic loading. In addition, the BOF slag has a complex pore structure and reasonable pore gradation, which enhances adhesion property between the rubber asphalt and BOF slag aggregates.
2. The incorporation method and grading type have significant influences on the durability of skid resistance. Generally, the steel slag mixtures with the volume blending method and SMA gradation can enhance the durability of the adhesive ability of the asphalt aggregate.
3. In terms of skid resistance test methods, the Wehner/Schulze Test and Aachen Polishing Machine testing methods can be used for asphalt mixtures and aggregates. Considering the polishing simulation and application range, the APM method is a more practical method.
4. Compared with dry conditions, the skid resistance of asphalt pavements under snow and ice conditions deserves more attention. The addition of steel slags can enhance the induction heating property of asphalt mixtures. Under microwave irradiation, the temperatures of asphalt mixtures were sharply raised, while the ice and snow of asphalt mixtures began to melt. This is an excellent way to ensure traffic safety in the cold winters.
5. The pavement will generate cracks due to the expansion of steel slags. This is harmful to the durability of the pavement. To limit the volume expansion of steel slag, some approaches have been applied, including natural aging or accelerating aging, which includes covering the steel slag surface with waterproof materials. Covering the steel slag surface is a very promising surface treatment approach for steel slags. If the TiO_2_ particles can be taken into consideration to be used as modified materials, more economic and environmental benefits will be obtained.
6. The long-term evolution of the skid-resistance property of BOF slag asphalt mixtures has a profound impact on the durability and service quality of the asphalt surface course, which needs to be focusing more attention on. Based on the differential polishing, the blends of BOF aggregate, and traditional aggregate can provide a new idea for the improvement of the anti-skid performance and durability of asphalt pavements. In view of this, more effective prediction models of life-cycle skid-resistance properties of BOF slag asphalt mixtures should be established, and exposed to the coupling effect of the variable environment and load.

For future research, the recommendations are proposed:

1. The mechanisms on how the difference of minerals can improve the polishing effect should be described.
2. Further combination with the surface macroscopic and microscopic decay test and the polishing evolution mechanism of BOF steel slag ultra-thin abrasion layer under long-term polishing should be completed.
3. A method to choose the suitable aggregate that can blend with BOF slag to gain the best differential polishing effect should be put forward.

## Figures and Tables

**Table 1:** Composition in %weight of basic oxygen furnace (BOF)-Steel obtained from different countries.

Country	MgO	Al_2_O_3_	SiO_2_	P_2_O_5_	CaO	MnO	Fe_2_O_3_
China [22]	5.19	3.25	19.24	1.41	42.70	1.77	24.55
USA [23]	12.7	2.3	9.3	0.3	11	1.8	26.2
Japan [24]	6.4	1.5	13.8	-	44.3	5.3	17.5
France [25]	5	2.5	13	1	40	6	29

**Table 2:** X-ray fluorescence (XRF) test results of different aggregates [22].

Chemical Composition of Different Aggregate (mass%)							
Item	MgO	Al_2_O_3_	SiO_2_	P_2_O_5_	CaO	MnO	Fe_2_O_3_
BOF	5.19	3.25	19.24	1.41	42.70	1.77	24.55
limestone	1.74	0.30	14.55	1.02	46.80	4.30	0.20
basalt	5.59	18.30	58.09	0.97	7.14	3.22	0.50

**Table 3:** Mineral phases in BOF slag [8].

Mineral Phase	α-Dicalcium Silicate	β-Dicalcium Silicate	γ-Dicalcium Silicate	Tri-calcium Silicate	RO Phase
Chemical Formula	(α-C_2_S)	(β-C_2_S)	(γ-C_2_S)	(C_3_S)	MgO-FeO-MnO

**Table 4:** Technical indicators of BOF slag and natural aggregates [30].

Properties	Units	BOF Slag	Basalt	Limestone	JTG F40-2004 *
Bulk Density	g/cm^3^	3.290	2.900	2.750	≥2.6000
Water Absorption	%	1.18	0.70	1.05	≤2.00
Los Angeles coefficient (LA)	%	13.1	14.9	22.0	≤28.0
Polished Stone Value (PSV)	%	57	49	44	≥42
Crush value	%	12.0	12.9	15.1	≤26.0

* JTG F40-2004 is Technical Specification for Construction of Highway Asphalt Pavements in China.

**Table 5:** The methods to control the volume expansion of steel slag.

Methods	Researchers
Covering steel slag surface	Chen [36], Ravichandar [37], Zhao [38].
Aging	Chen [35], Bocci [39], Shu [40], Zhao [41].
Using acidic compound to react with steel slag	Ding [42], Teir [43].

**Table 7:** The incorporation methods of aggregate in asphalt mixture [67,68,69,70,71].

Aggregate Type	Density Difference (%)	MaximumSize (mm)	Mixtures Type	Incorporation Method
BOF with Gneiss [68]	18	4.75	Surperpave-12.5	In volume
Ceramsite with Basalt [69]	50	13.2	SMA-13	Volume mass conversion
BOF with Basalt [70]	15-20	13.2	AC-13, SMA-13	Volume mass conversion
Limestone with Basalt [71]	5	16	AC-13, SMA-13	In mass
Limestone with Basalt [72]	3	16	AC-13, SMA-13	In mass

AC-XX refers to Asphalt mixtures. SMA-XX refers to Stone Mastic Asphalt mixtures. Surperpave refers to Superior Performing Asphalt Pavements.

**Table 8:** Skid resistance of BOF slag asphalt mixtures with different gradation.

Reference	Aggregate	Gradation	Indicators	Device	Results	JTG D50-2017
Xu [79]	BOF Basalt	AC-13 AC-25	TD BPN_20_	SP BPT	0.64(13), 0.69(25) 75.2(13), 79.3(25)	BPN_20_ ≥ 45 TD ≥ 0.55 mm DF_60_ ≥ 54
Wang [77]	BOF Basalt	SMA-10 SAC-10	TD	SP	084 0.98	
Niu [70]	BOF Basalt	AC-13 SMA-13	DF_60_	DFT	0.58(A), 0.57(S)	
Xue [30]	BOF Basalt	SMA-13	TD BPN_20_	SP BPT	0.8 62	
Xie [22]	BOF Basalt	AC-16C	TD BPN_20_	SP BPT	0.66, 68.3	

SP is Sand Patch test, BPT is British Pendulum tester, DFT is Dynamic Friction tester. JTG D50-2017 is Specifications for Design of Highway Asphalt Pavement in China.
